# Co-culture: A quick approach for isolation of street rabies virus in murine neuroblastoma cells

**DOI:** 10.14202/vetworld.2015.636-639

**Published:** 2015-05-21

**Authors:** A. Sasikalaveni, K. G. Tirumurugaan, S. Manoharan, G. Dhinakar Raj, K. Kumanan

**Affiliations:** 1 Department of Animal Biotechnology, Madras Veterinary College, Tamil Nadu Veterinary and Animal Sciences University, Chennai - 600 007, Tamil Nadu, India; 2 Dean, Faculty of Basic Sciences, Tamil Nadu Veterinary and Animal Sciences University, Madhavaram Milk Colony, Chennai - 600 051, Tamil Nadu, India

**Keywords:** co-culture, isolation, fluorescent-antibody test, murine neuroblastoma, rapid tissue culture infection test

## Abstract

**Background:**

Laboratory detection of rabies in most cases is based on detection of the antigen by fluorescent antibody test, however, in weak positive cases confirmative laboratory diagnosis depends on widely accepted mouse inoculation test. Cell lines like neuroblastoma have been used to isolate the virus with greater success not only to target for diagnosis, but also for molecular studies that determine the epidemiology of the circulating street rabies strains and in studies that look at the efficiency of the developed monoclonal antibodies to neutralize the different rabies strains. Due to the recent issues in obtaining ethical permission for mouse experimentation, and also the passages required in the cell lines to isolate the virus, we report herewith a co-culture protocol using the murine neuroblastoma (MNA) cells, which enable quicker isolation of street rabies virus with minimum passages.

**Objective:**

This study is not to have an alternative diagnostic assay, but an approach to produce sufficient amount of rabies virus in minimum passages by a co-culture approach in MNA cells.

**Materials and Methods:**

The MNA cells are co-cultured by topping the normal cells with infected cells every 48 h and the infectivity was followed up by performing direct fluorescent-antibody test.

**Results:**

The co-culture approach results in 100% infectivity and hence the use of live mouse for experimentation could be avoided.

**Conclusion:**

Co-culture method provides an alternative for the situations with limited sample volume and for the quicker isolation of virus which warrants the wild type strains without much modification.

## Introduction

Rabies virus is a highly neurotropic virus that affects all warm-blooded animals and transmission of this disease occurs mainly through dog bite in India. Even though the decision to withhold post-exposure vaccination following an animal bite does not depend on the results of a laboratory diagnosis a single test that is widely accepted is the fluorescent antibody test (FAT) when performed in a certified laboratory with high-quality reagents [1,2]. This FAT test can also result in false-negative results even though they are not common [3]. Hence, due to the greater significant impact of the laboratory results, small quantities of the virus in very weakly FAT positive samples are confirmed by the mouse inoculation test (MIT) [4]. Due to the delayed results provided by MIT and the non-availability of bio-safety facility this method is more restricted to institutions with containment facility and also for epidemiological purposes. In the recent years, attempts are always directed to find suitable alternatives to the use of live animals. Post-exposure prophylaxis for rabies uses a combination of rabies vaccine and anti-rabies immune globulin (RIG) with the latter being obtained from rabies vaccinated human donors (HRIG) or horses (ERIG) which has been a shortage in most of the countries. Attempts are on the way in many labs to generate Mab cocktail that are capable of neutralizing rabies virus strains, which will provide an alternate to RIG [5-8].

In view of the above two situations, sensitive cell lines would not only be less expensive, but also provide repeatable and quicker results [9-11]. Methods to amplify the street virus in fewer passages will also be very much useful. Hence, in this study, we attempted a co-culture method for amplification of street rabies virus samples in MNA samples with the hypothesis that it would not only allow rapid isolation of street rabies virus, so that the virus is available can be used for a wide variety of downstream applications including molecular epidemiology and neutralization studies.

## Materials and Methods

### Ethical approval

This work involved *in-vitro* processing the samples for isolation of the virus from brain samples obtained at post-mortem in murine neuroblastoma cells and hence did not require ethical approval.

### Samples

The brain samples used in this study were collected at post mortem from rabies suspected cases that were referred to the Department of Veterinary Pathology, Madras Veterinary College, Chennai - 600 007. The anti-nucleocapsid conjugate (Bio-Rad Laboratories) was used for detecting the rabies antigen from both the brain sample and the infected cells. All the positive samples were stored at −80°C until use. The murine neuroblastoma (MNA) cells were used from the Rabies Units of the Department of Animal Biotechnology, Madras Veterinary College, Chennai - 600 0077. The cells were grown in Dulbecco’s Modified Eagle Medium supplemented with 10% fetal bovine serum (FBS) and antibiotic stock (Invitrogen, CA).

### Techniques

**Figure-1:**
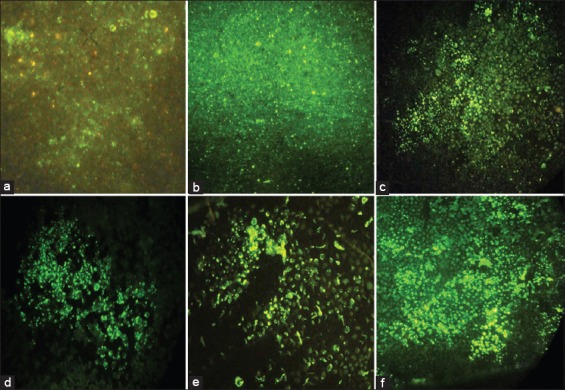
Co-culture method for quicker isolation of street rabies virus in murine neuroblastoma cells, (a). Direct fluorescent antibody test (FAT) on the dog brain sample, (b). On the mice brain following MIT on 12^th^ day Isolation of street rabies virus in MNA cells, (c) Direct FAT on the neuroblastoma cells, (Passage 2), (d). Direct FAT on the N2a cells (Passage 3), (e). Direct FAT on the N2a cells (Passage 4), (f). Direct FAT on the N2a cells (Passage 5).

Impression smears accompanied all the samples, which were screened by direct FAT as per standard procedures employing the anti-nucleocapsid conjugate (Figure-1a and b). Five brain samples that tested positive by direct FAT were selected for isolation of the street rabies virus employing the co-culture method in MNA cells. A 1% suspension of each of the brain samples were made in phosphate buffered saline with 2.5% FBS. The brain suspensions were centrifuged at 1500 rpm for 5 min and the supernatant used to infect the MNA cells and also for MIT. All the culture protocols in this study were performed in 12 well cell culture plates with two milliliters of the medium and 1 × 10^6^ cells per well.

For the co-culture method to infect the MNA, 1 × 10^6^ cells were seeded to a single well in a 12 well plate with 2 ml of medium and incubated at 37°C for 6 h. After 6 h, the medium from the well was removed leaving 0.5 ml of medium to which 0.5 ml of the 1% brain suspension was added and the cells incubated at 37°C for 1 h. The wells were topped up with 1 ml of medium and incubated for 48 h. MIT was also performed as per standard procedures with 30 µl of the brain suspension that was used to infect the MNA cells for comparing the efficiency of the isolation. On the same day of infecting the cells, we prepared another well with MNA at a density of 0.25 × 10^6^ cells in 2 ml of the media in the same plate. 48 h following infection, the medium from the infected wells was removed, the cell sheet briefly trypsinized to suspend the cells in 2 ml of media. From this 0.5 ml of cell suspension was added to the new well (0.25 × 10^6^ cells) that was prepared on the 1^st^ day and incubated for 48 h. Every time when cells were infected by the brain suspension or by transfer of the infected cells, MNA cells at a density of 0.25 × 10^6^ cells were prepared in a separate well to be used for the next passage after 48 h. This procedure of co-culture of the infected cells with the normal cells were performed every 48 h until the 5^th^ passage (10 days in total).

## Results

The progression of the infection during the co-culture was followed by conducting a direct FAT on the infected cells. In all the five samples tested by this co-culture method, the infectivity increased after each passage with 100% infectivity in the MNA cells by the fifth passage (Figure-1c-f). However, the same samples used for MIT had variability in the number of days (10-15 days) required for death of the inoculated mice and the presence of the virus in the brain samples were also confirmed by direct FAT. Even though 100% infectivity could be observed after five passages, we could detect reasonable viral load by FAT as early as second passage (4 days) thus being more advantageous than the MIT. Sacrificing the mice inoculated at the same time (on the 5^th^ day) for MIT with the street virus did not reveal any fluorescence in the brain impression smears (Figure-1b).

**Figure-2:**
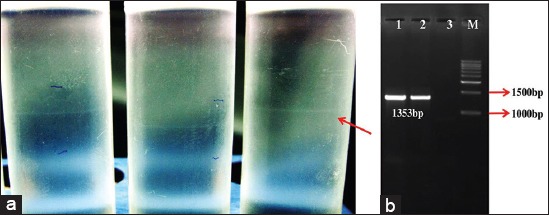
Purification of the passaged street rabies virus following co-culture approach and confirmation by polymerase chain reaction targeting the N gene, (a). Purification of the neuroblastoma propagated street rabies from dog brain sample (arrow shows the viral band), (b). Amplification of the full-length nucleoprotein of rabies virus by reverse transcription polymerase chain reaction to confirm the virus the primer sequence reported by Jayakumar *et al*., 2006 was used to amplify the full-length N gene. Lane M: 1 Kb DNA ladder, 1: Test sample, 2: Positive control, Lane 3: Negative control.

The infected cells from the 5^th^ passage were transferred to a 125 cm^2^ flask and incubated for 96 h. The cell culture supernatant from the flask was processed for crude pelleting and subsequence purification of the virus by gradient centrifugation (Figure-2a). RNA was extracted from the purified band and we performed reverse transcription polymerase chain reaction to amplify the full-length nucleoprotein gene [12] and to confirm virus (Figure-2b). Rapid tissue culture infection test (RTCIT) has really reduced the unpredictable and the delay that is usually associated with MIT. In addition, the rabies tissue culture infection test is also easy to perform, reliable, and cost effective than the MIT.

## Discussion

Tissue cultures have reported to be successful for the propagation of rabies virus since 1913 [13,14]. Among the cell types, baby hamster kidney (BHK-21), chick embryo related, and MNA cells being used as suitable *in-vitro* host system for isolation of street virus [15-17] with MNA being highly preferable due to its high sensitivity [17-19]. Characterization of virus like particles and purification of viral proteins were also reported effective with cell culture [20-23] and for the characterization of monoclonal antibodies (Mabs) [24,25]. India being a country endemic for rabies, studies on the local isolates is very much needed to understand more about the epidemiology of this disease. In addition in most of the cases, the samples that are being submitted to the laboratory for confirmation is not only very little to warrant propagation of the virus, but also requires a sensitive system. Even though previous reports have indicated good sensitivity of MNA cells for use in RTCIT with the fluorescence evident in the infected cells by around 72-96 h, the percentage of infected cells is very much lower when used for street rabies viruses. The percentages of infected cells in RTCIT for street rabies strains being lower need a mechanism to enhance the proportion of infected cells. In this context, a co-culture based method of exposing infected cells with normal cells have been reported for successful isolation and quicker propagation of viruses [26-29]. Our co-culture method of propagating the infected MNA cells with the non-infected cells every 48 h not only resulted in increasing the infectivity of the cells which is evident from the percentage FAT positive cells tracked through the course infection, but also helps in easy purification of the street virus sample in a shorter period of time.

## Conclusion

Thus, this method of co-culture provides a good option for quicker virus isolation will help in producing enough virus material that could be used to characterize street stains as a part of molecular epidemiology studies or in laboratories that look at neutralization efficiency of Mabs on street rabies virus strains. In addition, this co-culture method could be a good alternative in situations where the sample collected is very limited (e.g., cerebrospinal fluid). The reported method is not to be claimed as an alternate methodology for diagnosis, but an approach which will enable quick amplification of the virus to be used in studies which warrant wild type virus strains without much modification as situations indicated above.

## Authors’ Contributions

KGT, GDR, KK, SM conceived the study. KGT, GDR, KK were involved in designing of the experiment. KGT and AS were involved in Conduct of the experiment, documenting the results, analysis, and manuscript writing.. All authors read and approved the final manuscript.
